# Supplementary material for: Biocompatible and Biodegradable Magnesium Oxide Nanoparticles with In Vitro Photostable Near-Infrared Emission: Short-Term Fluorescent Markers
Source: Nanomaterials (Basel). 2019 Sep 23;9(10):1360. doi: 10.3390/nano9101360 (PMC6835516; doi:10.3390/nano9101360)
Supplement: Supplementary file 1 [file nanomaterials-09-01360-s001.pdf]

Supplementary Information

Biocompatible and biodegradable magnesium oxide nanoparticles with in vitro photostable near-infrared emission: short-term fluorescent markers

Asma Khalid,[a,b] Romina Norello,[a] Amanda N. Abraham,[b] Jean-Philippe Tetienne, [a] Timothy J. Karle, [a] Edward W. C. Lui,[c] Kenong Xia,[c] Phong A. Tran,[d,e] Andrea J. O'Connor,[e] G. Bruce Mann, [f,g] Richard de Boer, [g] Yanling He,[h] Alan Man Ching Ng,[h] Aleksandra B. Djurisic,[i] Ravi Shukla,[b] and Snjezana Tomljenovic-Hanic [a]

[a] School of Physics, University of Melbourne, Parkville VIC 3010, Australia
[b] School of Science, Engineering and Health, RMIT Univeristy, Melbourne VIC 3000, Australia
[c] Department of Mechanical Engineering, University of Melbourne, Parkville VIC 3010, Australia
[d] Faculty of Science and Engineering, Queensland University of Technology, Brisbane QLD 4000, Australia
[e] Department of Chemical and Biomolecular Engineering, Particulate Fluids Processing Centre, Melbourne, VIC 3010, Australia
[f] The Department of Surgery, University of Melbourne, Parkville VIC 3010
[g] The Breast Service, Victorian Comprehensive Cancer Centre, Parkville VIC 3052
[h] Department of Physics, Southern University of Science and Technology (SUSTech), Shenzhen, CN 518055 China
[i] Department of Physics, University of Hong Kong, Pokfulam Road, Hong Kong
*snjezana.thanic@unimelb.edu.au

S1. TEM images of MgO NPs

High resolution TEM images are obtained for individual MgO nanoparticles. An accelerating voltage of 200 kV is used to collect images at high magnifications of 10,000× to 50,000×. For commercial MgO, spherical nanoparticle shape is observed with a size distribution of 152±4 nm to 760 ± 80 nm for the individual NPs, as can be seen by in Figure S1 (a), (b). For ball milled MgO, irregular shaped crystals are observed with a relatively narrow size distribution ranging from 50±4 nm to 120±10 nm, as shown in Figure 1(b).

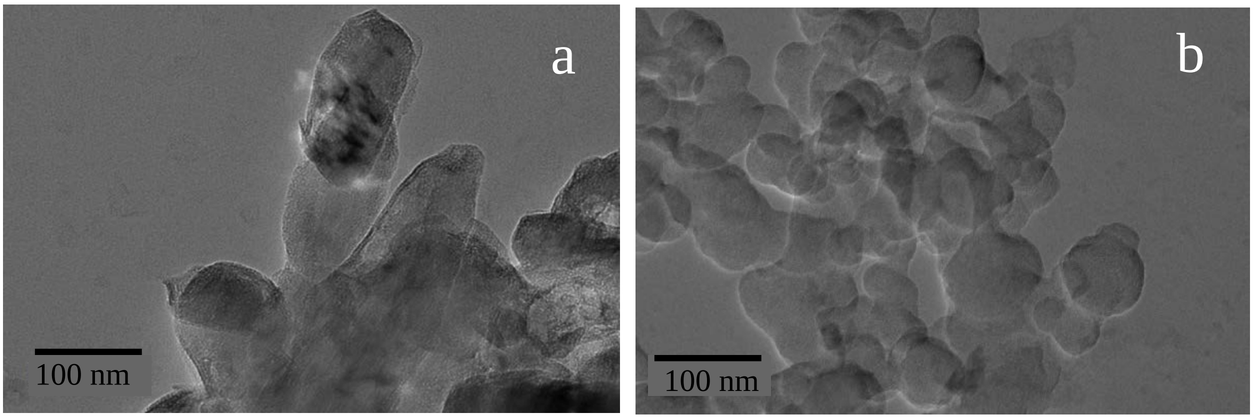

Figure S1: TEM images of (a) commercial MgO nanoparticles and (b) 4000 rpm pellet of ball-milled MgO nanoparticles.

S2. Lifetime measurements

The lifetime measurements were performed for both commercial and ball-milled MgO NPs. A typical fluorescence decay trace is shown in Fig. S2, along with the instrument response. The traces from the NPs were found to be well fitted using a bi-exponential function (see red line in Fig. S2 (a)). Figs. 2S (b) and (c) show the histograms of the two time constants τ_1 and τ_2, respectively, constructed from a set of about 20 NPs of each type. In both types, there is a fast decay component, with τ_1=1.5-3 ns, accompanied by a slower decay, τ_2=8-20 ns. This bi-exponential behaviour suggests a two-state fluorescent system, which could arise either from a single defect or from two distinct types of defects contained in the same NP. The fast decay shows a relatively similar distribution for the ball-milled and commercial NPs (Fig. S2 b), however the slow component differs significantly for the two types (Fig. S2 c), with τ_2 being shorter for the ball-milled NPs (10 ns on average) than for the commercial

NPs (15 ns). Moreover, the associated weight $A_2/(A_1+A_2)$ is larger in the ball-milled NPs (0.38 against 0.10 on average, see Fig. S2 (d)). Consequently, on average the slow component is responsible for 74% of the total fluorescence signal for the ball-milled NPs, against only 45% for the commercial NPs. Although differences in shape and size of the two types of NPs may play a role, we speculate that this discrepancy is a result of the different nature of the emitters in the two types, which from our spectral measurements (section 3.4) are believed to be mostly V^{2+} substitutional defects in the commercial NPs and Cr^{3+} substitutional defects in the ball-milled NPs. In future studies, more insight could be gained on the various defects present via correlative lifetime/spectral measurements.

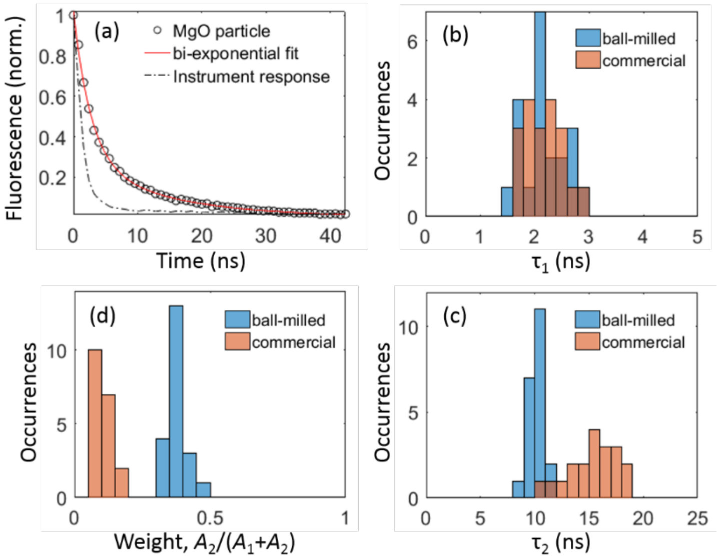

Fig. S2: (a) Example fluorescence decay trace measured from a single MgO (ball-milled) particle. The red line is a bi-exponential fit with time constants τ_1 and τ_2, and respective amplitude A_1 and A_2. The dotted-dashed line shows the instrument response. (b,c) Histograms of τ_1 (b) and τ_2 (c) obtained from measurements on about 20 particles of the commercial type (red) and ball-milled type (blue). (d) Histogram of A_2, relative to the total amplitude $A_1+A_2=1$.

S3. Cell viability

Bright field images of the PC-3 cells (Figure 6a), taken after 12 h exposure to the MgO nanoparticles show normal cellular morphology. The cells also exhibit polarisation, which is the presence of two to three processes emanating from the central body of the cell. This suggests the cells are capable of active migration, and further indicates normal cellular functioning. The 24 h toxicity studies, however, indicate that the MgO nanoparticles lead to a steady decline in cell viability from 62.5 µg/mL upwards, as shown in Figure S3. The effect of the MgO nanoparticles will have to be further investigated on an array of cancer cell lines to best determine their anti-cancer potential.

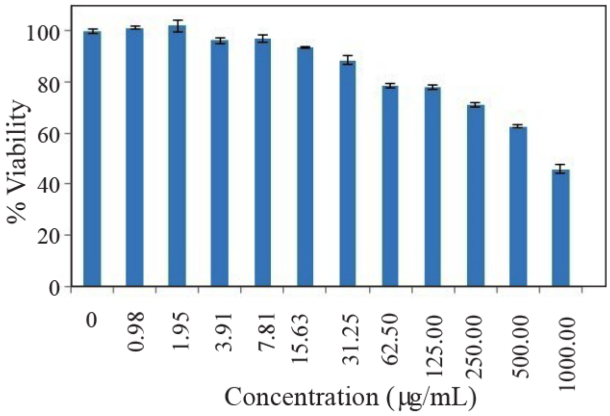

Figure S3: Cell viability of prostate cancer (PC-3) cells with MgO nanoparticles recorded for 24 h.

S4. Dynamic light scattering (DLS) of incubated MgO NPs

The y axis represents the intensity of light scattered by the particles. Since larger particle scatter more light hence a larger peak is observed for icron sized particles. The intensity values do not represent the number of particles and it is possible that the smaller sized particles are present in the same quanity as the bigger agglomerates.

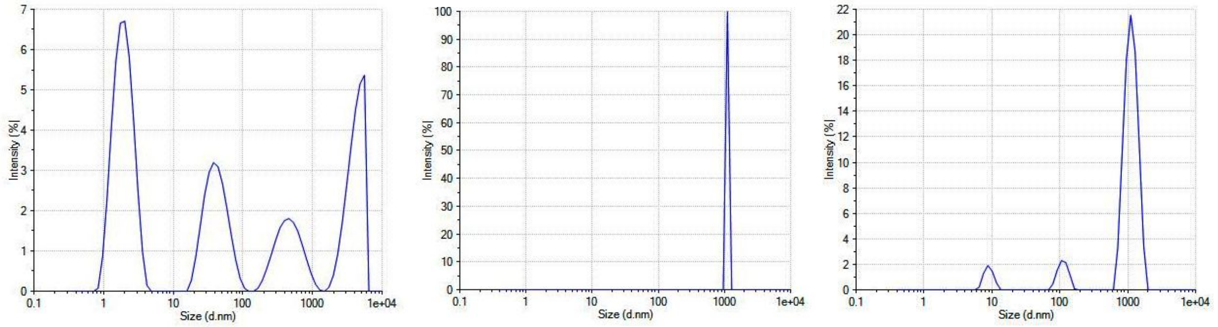

Figure S4: DLS size distribution of commercial MgO NPs incubated in water (left), PBS (middle) and culture medium (right) after 24 h. The y-axis represents the amplitude of scattered light which is higher for larger particles.

Hence the MgO NPs are considered stable in PBS+10% FBS, under cell culture conditions, in comparison to the MgO NPs in PBS alone. Extensive aggregation was observed in PBS, however in the presence of 10% FBS, protein corona formation was seen after 24 h. This is a commonly observed through the increase in hydrodynamic size of the NPs, as we similarly observed with the MgO NPs.
